# Different Therapeutic Effects of Transcranial Direct Current Stimulation on Upper and Lower Limb Recovery of Stroke Patients with Motor Dysfunction: A Meta-Analysis

**DOI:** 10.1155/2019/1372138

**Published:** 2019-11-16

**Authors:** Xi Bai, Zhiwei Guo, Lin He, Long Ren, Morgan A. McClure, Qiwen Mu

**Affiliations:** ^1^Department of Radiology and Imaging Institute of Rehabilitation and Development of Brain Function, The Second Clinical Medical College of North Sichuan Medical College Nanchong Central Hospital, Nanchong, China; ^2^Department of Radiology, The Fifth People's Hospital of Nanchong, Nanchong, China; ^3^Department of Radiology, Peking University Third Hospital, Beijing, China

## Abstract

**Objective:**

To explore the effects of transcranial direct current stimulation (tDCS) on the motor recovery of stroke patients and the effect differences between the upper limb and lower limb.

**Methods:**

Randomized control trials published until January 2019 were searched from PubMed, Embase, ScienceDirect, and Cochrane Library databases. The standardized mean difference (SMD) with 95% confidence interval (CI) was estimated separately for upper and lower limb motor outcomes to understand the mean effect size.

**Results:**

Twenty-nine studies with 664 subjects were included in this meta-analysis. The overall analyses of tDCS demonstrated significant effect size both for the upper limb (SMD = 0.26, *P* = 0.002) and the lower limb (SMD = 0.47, *P* = 0.002). Compared with acute and subacute stroke patients, chronic stroke patients obtained significant effects after tDCS (SMD = 0.25, *P* = 0.03) in upper limb function. Furthermore, both anode and cathode stimulations produced significant effect size for stroke patients after ≤10 sessions of tDCS (anode: SMD = 0.40, *P* = 0.001; cathode: SMD = 0.79, *P* < 0.0001) with >0.029 mA/cm^2^ of density (anode: SMD = 0.46, *P* = 0.002; cathode: SMD = 0.79, *P* < 0.0001). But for lower limb function, more prominent effects were found in subacute stroke patients (SMD = 0.56, *P* = 0.001) with bilateral tDCS (SMD = 0.59, *p* = 0.009).

**Conclusion:**

tDCS is effective for the recovery of stroke patients with motor dysfunction. In addition, upper limb and lower limb functions obtain distinct effects from different therapeutic parameters of tDCS at different stages, respectively.

## 1. Introduction

Stroke, a kind of cerebral blood circulation disorder disease which can cause neurological deficits, is one of the leading causes of disability in the world's elderly population [1]. Studies have predicted that stroke will occupy an undeniable proportion of the global burden of disease by 2020 reaching to 6.2% [2]. It is, therefore, very necessary to find a useful way to recover the motor function of paretic limb after stroke. The interhemispheric competition mechanism shows a reciprocal inhibition of the neural activity between bilateral hemispheres in a healthy brain, which is realized by the transcallosal fibers [3]. The balance is broken after a unilateral stroke resulting in excessive excitation of the unaffected hemisphere and increased inhibition to the affected hemisphere [4]. Therefore, rebalance of the brain is the key for the recovery of function [5]. In addition to competition mechanism, a recent, bimodal balance-recovery model has been proposed which holds that the competition and vicariation model combined to regulate the balance between the hemispheres [6].

Conventional therapies including constraint-induced movement therapy [7, 8], robotic training [9], and occupational therapy [10] play an important role for the recovery of motor function after stroke, but these physiotherapies have some limitations which make researchers intend to seek more effective ways to boost limb function recovery by reinforcing cortical plasticity [6]. Noninvasive brain stimulation has been prevalent in recent years. These mainly include tDCS and repetitive transcranial magnetic stimulation (rTMS). Compared with rTMS and traditional treatment methods, tDCS has the characteristics of feasibility, cost-effectiveness, convenience, etc. Also, a review [11] and two meta-analyses [12, 13] have indicated that tDCS is safe and tolerable. Up to now, a large number of studies on tDCS applied to neurological diseases have been published, such as stroke [14], depression [15, 16], and Parkinson's disease [17]. Noninvasive tDCS regulates motor cortex excitability mainly by influencing the polarity of the membrane [18]. Anode increases excitability and cathode reduces excitability [19]. Therefore, when the anode is applied to the ipsilesional cerebral hemisphere or the cathode to the contralesional hemisphere, the balance between the interhemispheres could be restored. Someone had described that cortical electrical stimulation combined with a motor training was better in improving motor function of the affected hand than a motor training alone [20]. And early intervention reduces the likelihood of functional disability [21].

Several reviews and meta-analyses had previously investigated the recovery of tDCS to upper limb motor function in survivors of stroke [20, 22–25]. However, opinions were varied. The majority of researches showed a beneficial effect to a paretic upper limb in stroke patients by tDCS [20, 24, 25], but in another two studies, one showed a tiny, nonsignificant effect on the impairment of upper extremity [22], and the other indicated that tDCS had no effect on the recovery of the arm function [23]. It was precisely because of the disagreements that further exploration was required. Furthermore, few articles discuss the specific parameters of tDCS to the upper limb. We would explore these in this article. Before our study, few reviews had researched the effect of tDCS on the lower limb function. Only one meta-analysis denoted that tDCS improved the lower limb muscle strength [26]. It is valuable to do something in lower limb function after stroke.

To sum up, the main purpose of this meta-analysis was to obtain different therapeutic effects of tDCS on upper and lower limb recovery of stroke patients with motor dysfunction. First, we need to confirm whether tDCS is beneficial to upper and lower limb function after first unilateral stroke and then to further explore the optimal parameters of tDCS and which stage of stroke is more effective by tDCS.

## 2. Methods

### 2.1. Search Strategy

We have searched clinical trials published before January 12, 2019, from PubMed, Embase, ScienceDirect, and Cochrane Library databases. The keywords were “tDCS” or “transcranial direct current stimulation,” “stroke,” or “cerebrovascular accident”.

### 2.2. Inclusion and Exclusion Criteria

In order to guarantee the quality of articles, we unified the inclusion criteria. The details were as follows: (1) all patients were adults (≥18 years) and were diagnosed with a first stroke; (2) the articles were focused on the effect of tDCS on the recovery of motor function in stroke patients; (3) the stimulation sites were located in the primary motor cortex (M1); (4) all experiments were randomized control trials including crossover and parallel design; (5) ≥5 patients were enrolled, and all control groups were sham tDCS; (6) all included articles were peer-reviewed and published in English; and (7) the results were measured with scales. The exclusion criteria were as follows: (1) patients who had other diseases that could cause motor dysfunction; (2) articles that had been published but did not provide raw data, such as reviews, meta-analysis, or case reports; (3) animal experiments; and (4) results that were not expressed as mean ± standard deviation or mean ± standard error, but as median or interquartile range.

### 2.3. Literature Quality Assessment

Two reviewers independently completed the methodological quality assessment of the included articles by using a modified checklist from Moher et al. [27]. If the opinions were inconsistent, a third reviewer reevaluated the articles and discussed with the two reviewers to reach an agreement. The specific criteria were as follows: (1) randomization; (2) blind procedure; (3) baseline data description; (4) control study; (5) dropout number; and (6) side effects. Randomization was recorded as “1” if the random distribution method was used in the study and “0” if not. For the blinded method, “0” represented the nonblinded procedure, and “1” and “2” represented the single and double-blind procedures, respectively. If baseline data was given, it was denoted as “1” and was denoted as “0” if not. The control design was recorded as “1” if the experiment was designed with a healthy control group, “2” with a patient control group, and “3” with both control groups. Dropout number and side effects were recorded as a number of events.

### 2.4. Data Extraction and Analyses

Two experienced reviewers completed the data extraction independently. The basic information included sample size, mean age, time of poststroke, study design, stimulation parameters (current density, session), stimulation site, and outcome measurements. If no change data was given in the article, the data of prestimulation and poststimulation was extracted. If only the graph was given in the article, we used the software of GetData Graph Digitizer 2.25 (http://getdata-graph-digitizer.com/) to get useful data.

An immediate effect was performed to investigate the curative effect on motor function recovery of stroke patients between real and sham tDCS. The following subgroup analyses were performed: polarity (anode vs. cathode vs. bilateral), stroke stage (acute vs. subacute vs. chronic), current density (≤0.029 mA/cm^2^ vs. >0.029 mA/cm^2^), and treatment sessions (≤10 sessions vs. >10 sessions).

Review Manager Software version 5.3 (Cochrane Collaboration, Oxford, England) was used to analyze data in this meta-analysis. The effect of tDCS was expressed as the standardized mean difference (SMD) with 95% confidence interval (CI). The heterogeneity was estimated by using Cochran's *Q* test and *I*^2^ test. If the *I*^2^ value was smaller than 50%, the fixed effects model was used; otherwise, a random effects model was used. View publication bias with a funnel plot. A statistically significant *P* value was set to 0.05.

## 3. Result

### 3.1. Literature Selection

**Figure 1:**
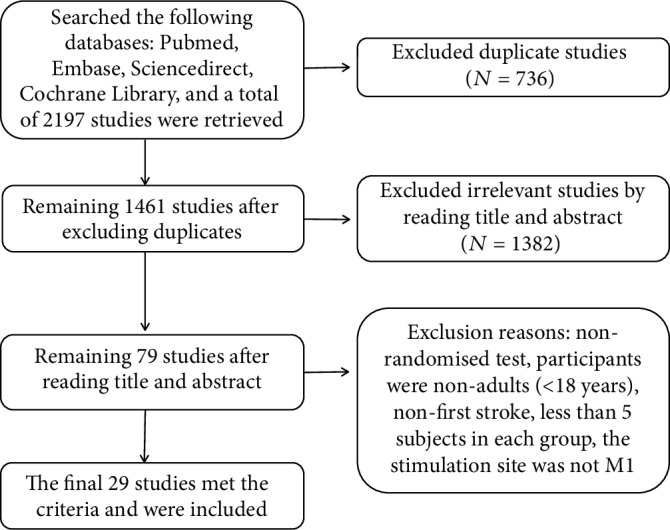
The specific flow chart of selection process.

By searching the four databases mentioned above, a total of 2,197 articles were found. Finally, only 29 studies were included in this meta-analysis based on the inclusion and exclusion criteria. The specific flow chart is shown in Figure 1.

### 3.2. Literature Characteristics

In the final included articles, stroke patients enrolled in two articles were in the acute phase [10, 28], 11 in the subacute phase, and 13 in the chronic phase. The remaining three papers recruited both subacute and chronic stroke patients. With regard to the experimental method, all the included studies were randomized control trials. As for the treatment parameters of tDCS, the current density ranged from 0.014 mA/cm^2^ to 0.28 mA/cm^2^. For sessions, at least one session up to 40 sessions of tDCS were performed. The stimulation sites of all included articles were M1. All included subjects suffered their first ever unilateral stroke. The main characteristics and the quality assessment of the included studies are shown in Tables 1 and 2, respectively.

### 3.3. Adverse Effect

A total of 11 articles showed side effects; three studies reported a tingling sensation under the electrodes [10, 28, 29]. Fleming et al. reported one patient quit due to a headache after the first intervention [30]. Triccas et al. mentioned one patient dropped out due to a skin reaction after receiving treatment and other side effects could be accepted by the remaining patients [31]. In the experiment of Kim et al., two subjects discontinued tDCS, one was due to headache and the other was because of dizziness [32]. Pain occurred in both groups in the study of Fusco et al. [33]. Light flashes, fatigue or warmth, etc. occurred in another four studies, but all subjects could endure and complete treatments. The remaining 18 articles either had not side effects or were not mentioned in the articles.

## 4. Results of Meta-Analysis

### 4.1. Overall Effect of tDCS

**Figure 2:**
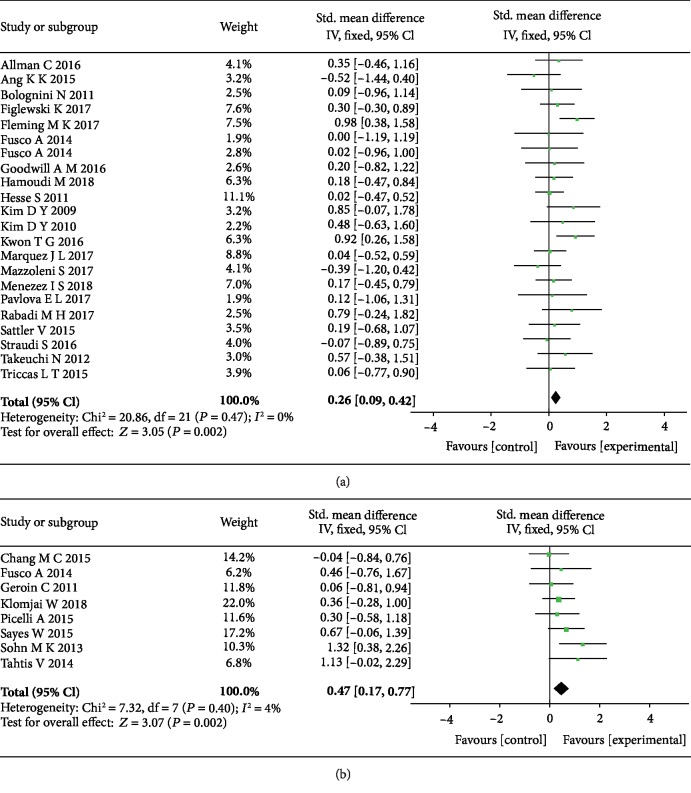
The forest plots of the overall effect of tDCS on upper (a) and lower (b) limb motor functions in stroke patients.

**Figure 3:**
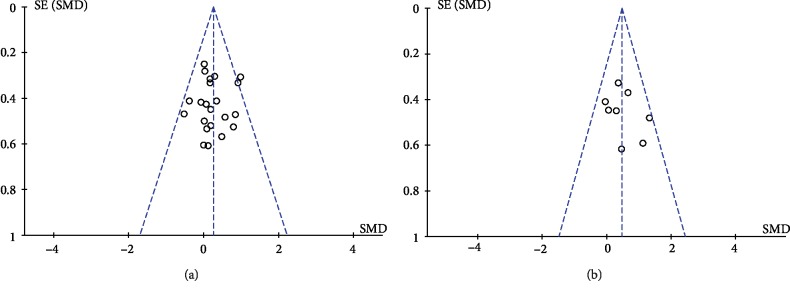
The funnel plots of the overall analysis of studies on upper (a) and lower limb (b) functions of stroke patients.

The pooled results showed that tDCS was effective to upper limb (SMD = 0.26, 95% CI: 0.09-0.42, *P* = 0.002, *I*^2^ = 0%) and lower limb (SMD = 0.47, 95% CI: 0.17-0.77, *P* = 0.002, *I*^2^ = 4%) function in stroke patients (Figure 2). The corresponding funnel plots above were approximately symmetrical and closed to the center line which indicated very small publication bias (Figure 3).

### 4.2. Upper Limb: Immediate Effect of tDCS to Different Stages of Stroke Patients

**Figure 4:**
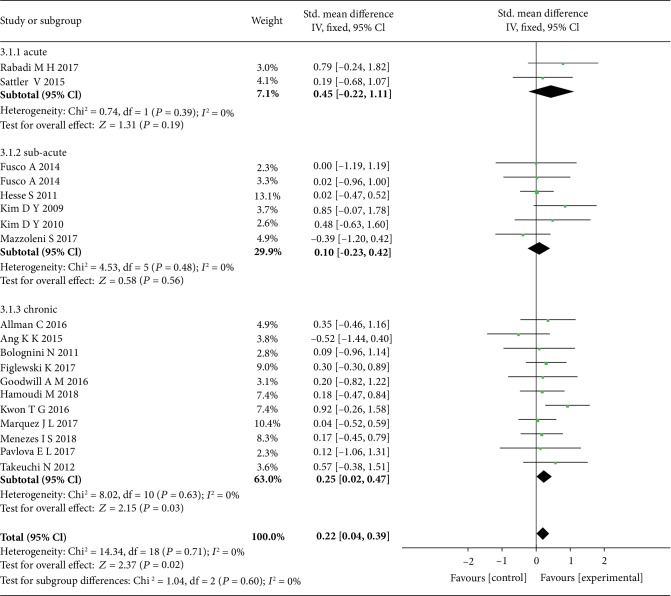
The forest plot shows more significant effect size of tDCS on chronic stroke patients with upper limb motor dysfunction than acute and subacute stroke patients.

According to the duration of stroke, the included studies were divided into the acute, subacute, and chronic groups. The comparison of the pooled results showed that the tDCS only revealed a significant effect (SMD = 0.25, 95% CI: 0.02-0.47, *P* = 0.03, *I*^2^ = 0%) in the patients of the chronic group (Figure 4).

### 4.3. Upper Limb: The Parameters of tDCS

**Figure 5:**
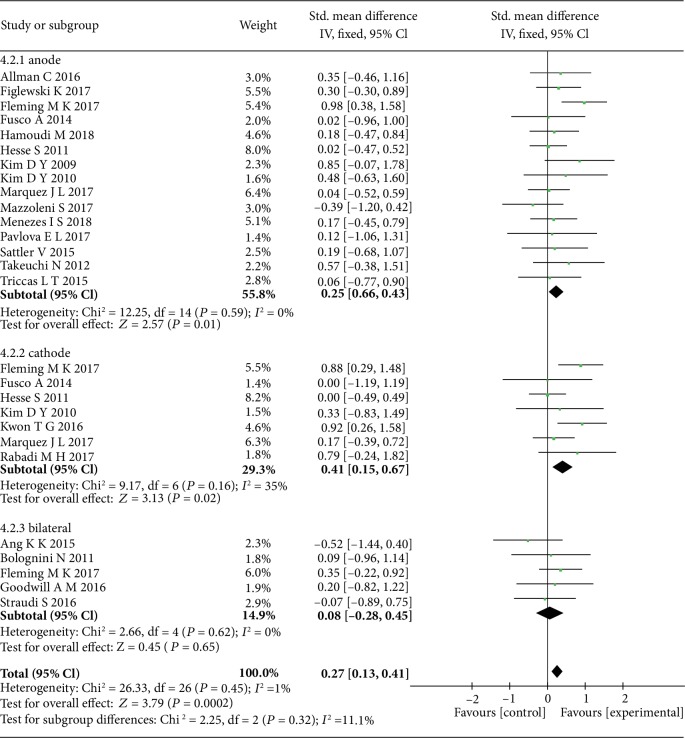
The forest plot shows more significant effect sizes of anode and cathode tDCS than bilateral tDCS on upper limb motor function in stroke patients.

The subgroup analysis of polarity showed that both the anode tDCS (SMD = 0.25, 95% CI: 0.06-0.43, *P* = 0.01, *I*^2^ = 0%) and cathode tDCS (SMD = 0.41, 95% CI: 0.15-0.67, *P* = 0.002, *I*^2^ = 35%) were significantly effective on upper limb function recovery (Figure 5). Therefore, further subgroup analyses of sessions and current density were conducted based on the anode and cathode tDCS studies separately.

**Figure 6:**
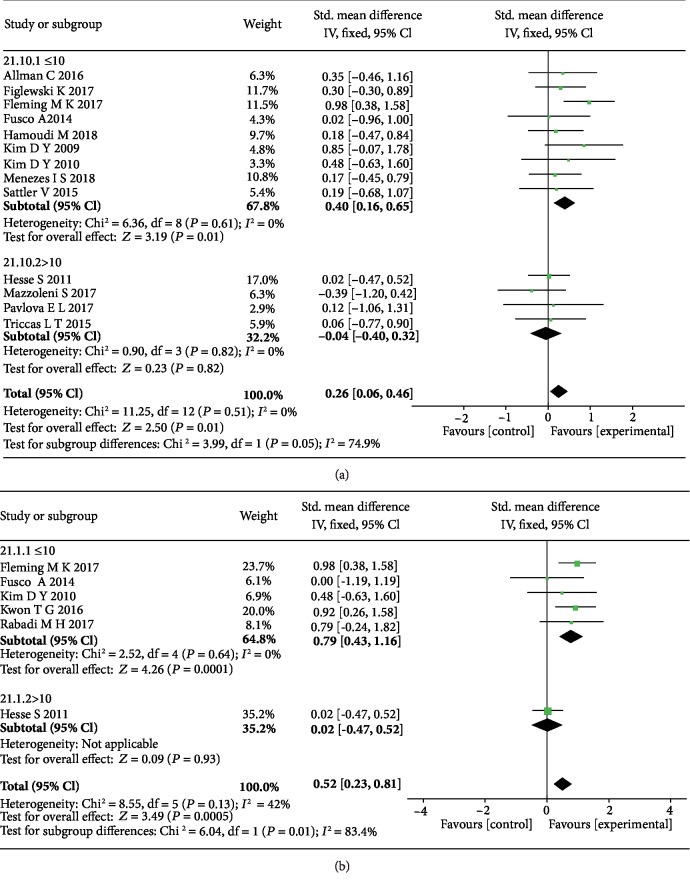
The forest plots of the subgroup analyses of treatment session of both anode (a) and cathode (b) tDCS show more significant effect sizes when ≤10 sessions on upper limb motor function in stroke patients.

The subgroup analysis of sessions revealed that the significant mean effect sizes were observed when ≤10 sessions of tDCS were given in both the anode tDCS (SMD = 0.40, 95% CI: 0.16-0.65, *P* = 0.001, *I*^2^ = 0%) (Figure 6(a)) and cathode tDCS groups (SMD = 0.79, 95% CI: 0.43-1.16, *P* < 0.0001, *I*^2^ = 0%) (Figure 6(b)).

**Figure 7:**
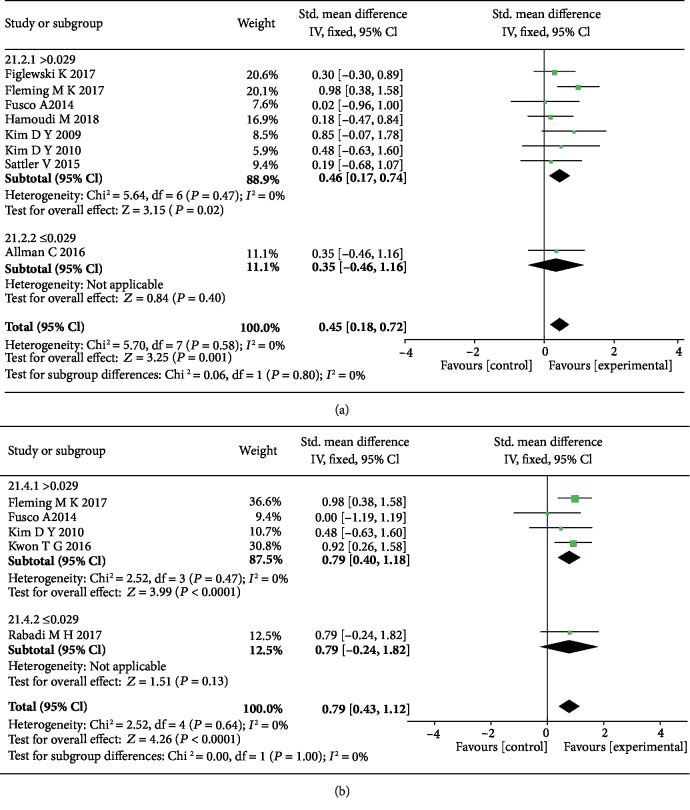
The forest plots of the subgroup analyses of stimulation density of both anode (a) and cathode (b) tDCS show more significant effect sizes when ≤10 sessions and the current density > 0.029 mA/cm^2^ on upper limb motor function in stroke patients.

For the stimulation current density, we observed that the mean effect sizes were more significant when the current density of tDCS was more than 0.029 mA/cm^2^ on the basis of ≤10 sessions also in both the anode tDCS (SMD = 0.46, 95% CI: 0.17-0.74, *P* = 0.002, *I*^2^ = 0%) (Figure 7(a)) and cathode tDCS groups (SMD = 0.79, 95% CI: 0.40-1.18, *P* < 0.0001, *I*^2^ = 0%) (Figure 7(b)).

### 4.4. Lower Limb: Immediate Effect of tDCS to Stroke Patients

**Figure 8:**
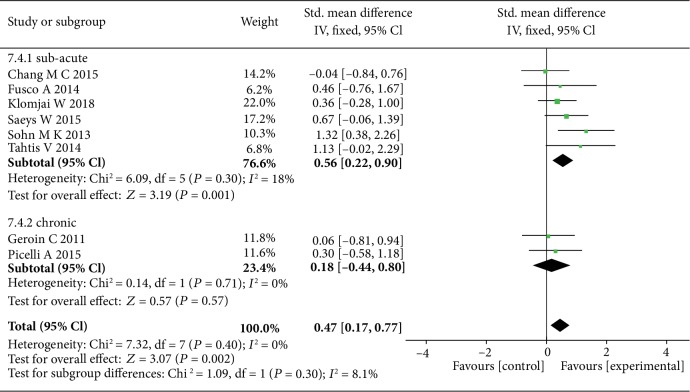
The forest plot shows more significant effect size of tDCS on subacute stroke patients with lower limb motor dysfunction.

**Figure 9:**
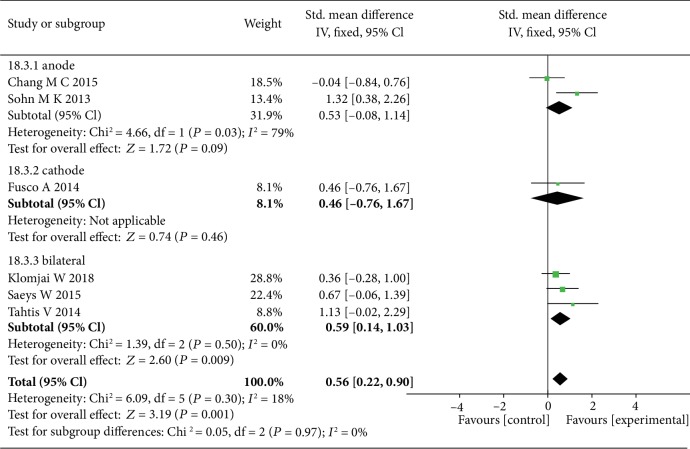
The forest plot shows more significant effect size of bilateral tDCS on lower limb motor function in subacute stroke patients.

The data of six studies were summarized and found that tDCS had a better significant effect on the recovery of lower limb function in sub-acute stroke patients (SMD = 0.56, 95% CI: 0.22-0.90, *P* = 0.001, *I*^2^ = 18%) (Figure 8), especially bihemispheric tDCS stimulation (SMD = 0.59, 95% CI: 0.14-1.03, *P* = 0.009, *I*^2^ = 0%) (Figure 9).

## 5. Discussion

Although many articles have proved that tDCS was beneficial to motor function recovery after stroke, few have analyzed the specific parameters. In this meta-analysis, we observed the positive effects of tDCS on the recovery of limb motor function after stroke. This was consistent with Edwardson et al. [34], but some differences existed between upper and lower limbs in tDCS parameters.

In terms of tDCS stimulation mode, we found that both the anode and cathode were beneficial to the recovery of upper limb motor function after stroke, which is in accordance with a previous review by Lüdemann-Podubecká et al. [25]. Two other papers had a similar conclusion that tDCS was beneficial to upper limb function in stroke patients [20, 24]. Although another meta-analysis reported that the effect of anode tDCS on stroke patients with motor dysfunction was not significant, it was positive for the motor recovery [19]. Furthermore, the increased corticomotor excitability was also observed in both stroke and healthy subjects in this study. Therefore, tDCS has a positive effect on the motor recovery of stroke patients, and its therapeutic effect may be related to the modulation of the neural activity between interhemispheres. The motor cortical excitability was imbalanced after stroke, due to the overactive unaffected hemisphere which increased the inhibition to the affected hemisphere [35]. The cathode or anode tDCS could promote the recovery of motor function by inhibiting the hyperactivity of the unaffected motor cortex or activating the injured motor cortex [36].

In further subgroup analysis on stroke stages, we found that the therapeutic effect of tDCS was better in chronic stroke patients than acute and subacute patients with upper limb disorder. This is consistent with the results in Jodie Marquez et al. [37]. Lüdemann-Podubecká et al. also held the same view that tDCS existed a positive effect in chronic stroke with upper limb dysfunction [25]. The excitability of the corticospinal is different in the subacute and chronic phases during stroke rehabilitation. Several functional magnetic resonance imaging studies of the brain have shown that the unaffected hemisphere is overactive after stroke. The hyperactivity leads to a less effect on the recovery of affected hand function in the subacute phase, but a better effect on the chronic phase [20, 38, 39]. However, the specific mechanism in this phenomenon was not very clear. It may be due to that after three to six months, neural recovery reached peak and remained stable [40, 41]. In addition, a study reported that short latency afferent inhibition (a marker of central cholinergic activity) was suppressed at the acute phase, but increased at six months, which was associated with better recovery of motor function [42].

One previous meta-analysis demonstrated that a positive dose-response relationship existed between current density and recovery of motor function after stroke [43]. This is in line with the results of our meta-analysis. Nitsche et al. found that the membrane depolarization or hyperpolarization induced by tDCS was determined by the current density. The larger the current density, the better the effect of tDCS [44]. Another systematic review and meta-analysis indicated that the efficacy of anode tDCS depended on the current density and duration of stimulation [19]. They observed that the effect of current density above 0.029 mA/cm^2^ was more significant than that below 0.029 mA/cm^2^. However, they subsequently found that the changes in corticospinal excitability caused by the current density of anode tDCS at 0.013 mA/cm^2^ were significantly larger than that at 0.029 mA/cm^2^ [45]. They believed that the current density at 0.013 mA/cm^2^ is sufficient to activate calcium channels and increase intracellular calcium content, which leads to neuron depolarization. But they also found that when the current density was above 0.029 mA/cm^2^, there was a direct relationship between the current density and the corticospinal excitability changes. This finding is partially in agreement with Nitsche et al. The difference could be explained in two aspects. On the one hand, the stimulation duration of this study was longer than that of Nitsche et al.'s. On the other hand, different electrode sizes were used in the studies of Bastani et al. (24cm^2^) and Nitsche et al. (35cm^2^), respectively. In addition, the higher the current density, the deeper the electrical field penetrated, which could change the excitability of the undamaged neurons. If the electrode size was too large, it not only stimulated the target region but also influenced the adjacent cortex. Moreover, there is a direct relationship between current density and side effects [45]. The accumulated toxic substance in sponge electrodes may also cause anodal skin lesions [40]. Therefore, an optimal but not maximum current density may exist, which could produce the best therapeutic effect for stroke patients. This is an issue that should be resolved in the future studies.

The same as the current density, the treatment session also influences the effect of tDCS. In this study, we found that the effect of tDCS ≤ 10 sessions on upper limb function recovery in stroke patients was significantly higher than that of other sessions both with anode and cathode stimulation. This coincided with the conclusion of Chhatbar et al., in which a dose-response relationship was not found in the number of session [43]. Lindenberg et al. also showed that the linear response was not necessarily existed between session and tDCS effect [46]. Therefore, it is not that the number of sessions the patient accepted, but the better the outcome will be. In terms of session, few researches have discussed it. Therefore, more studies are needed to explore this question further.

Prior to this study, a few meta-analyses were published on the recovery of lower extremity motor function after stroke. In our study, we found that bilateral tDCS significantly improved lower limb function in subacute stroke, as reported in the studies of Saeys et al. and Tahtis et al. They both suggested that bihemispheric stimulation made a good effect to lower limb function in subacute stroke [47, 48]. Bilateral tDCS reduces the excitability of the contralesional cortex and improves that of the ipsilesional cortex by placing anode on the affected side and cathode on the unaffected side. Therefore, bilateral tDCS is more conductive to reaching balance between the cerebral hemispheres. Feng et al. and Vines et al. also suggested that tDCS stimulated motor cortex simultaneously which was ideal to improve motor function [36, 49]. A suggestion about tDCS montage was put forward, prompting that bilateral stimulation was better than anode or cathode alone [11, 49, 50]. In a recent meta-analysis of lower extremities, tDCS performed well in mobility and muscle strength in patients with subacute stroke, but poorly in walking speed and balance function [26]. This was consistent with a part of our results. In our study, we also analyzed the polarity of tDCS but did not carry out a more detailed subgroup analysis. Several papers have mentioned that tDCS could increase the neural plasticity, improve motor function during spontaneous recovery, and reach maximum recovery in the first three months [34, 51]. This coincides with our results; that is, tDCS has a better therapeutic effect on subacute stage.

### 5.1. Limitation

There are still many details that could be improved in our meta-analysis. For example, only English studies were included in this study that may lead to publication bias. We did not limit the lesion of stroke (cortical or subcortical), nor did we summarize the follow-up results. In addition, most of the included studies enrolled ischemic stroke patients; whether these results are suitable for patients with hemorrhagic stroke still needs to be discussed further. In future studies, these mentioned aspects should be paid more attention.

## 6. Conclusion

tDCS is effective for the recovery of stroke patients with limb dysfunction after the first unilateral stroke, but the optimal parameters of tDCS for the upper and lower limbs are different. tDCS has a great impact on the recovery of upper limb function in chronic stroke patients. In addition, stroke patients with upper limb hemiplegia recover better by using anode or cathode tDCS with above 0.029 mA/cm^2^ current density and ≤10 sessions of treatment. But for the recovery of lower limb function, subacute stroke patients benefit more from bilateral tDCS.

## Figures and Tables

**Table 1:** Characteristics of the included studies.

Study	*N*	Mean age (y)	Time of poststroke	Study design	Current density (mA/cm^2^)	Session	Stimulation site	Out measure
Kim et al. 2009	10	62.8 ± 13.2	6.4 ± 3.2 w	Crossover	0.04	1	M1	FA
Menezes et al. 2018	20	56.6 ± 12.3	5.7 ± 5.7 y	Crossover	NA	1	M1	Pinch strength
Tahtis et al. 2014	14	61.9 ± 12.9	22.6 ± 9.4 d	Parallel	0.08	1	M1	TUG
Bolognini et al. 2011	14	46.7 ± 14.1	35.2 ± 26.4 m	RCT	0.057	10	M1	FMM
Kwon et al. 2016	20	58.6 ± 8.4	42.0 ± 37.5 m	Crossover	0.08	1	M1	Movement time
Rabadi et al. 2017	16	62 ± 9	6.4 ± 3.2 d	RCT	0.029	10	M1	ARAT
Pavlova et al. 2017	11	60.7 ± 11.4	5.5 ± 9.9 y	RCT	0.014	40	M1	FMA-UE
Mazzoleni et al. 2017	24	72.63 ± 10.78	25.38 ± 12.76 d	RCT	0.057	30	M1	FMA-UE
Figlewski et al. 2017	44	A: 60 ± 11 S: 61 ± 10	A: 9 (3-35) m S: 7 (3-36) m	RCT	0.043	9	M1	WMFT FAS
Takeuchi et al. 2012	18	A: 57 ± 10.2 S: 64 ± 5.8	A: 62.0 ± 33.2 m S: 71.9 ± 51.0 m	RCT	0.04	NA	M1	Pinch force
Marquez et al. 2017	25	64.28 ± 11	80.4 ± 43.5 m	Crossover	0.029	NA	M1	JTT
Straudi et al. 2016	23	58.2 ± 14.4	58.6 ± 52.2 w	RCT	0.029	10	M1	FMA-UE
Kim et al. 2010	18	A: 55.3 ± 16.4 C: 53.6 ± 14.9 S: 62.9 ± 9.2	A: 34.0 ± 27.1 d C: 19.4 ± 9.3 d S: 22.9 ± 7.5 d	RCT	0.08	10	M1	FMA
Goodwill et al. 2016	15	56.9 ± 12.3	4.5 ± 3.3 y	RCT	0.06	9	M1	MAS
Hesse et al. 2011	95	A: 63.9 ± 10.5 C: 65.4 ± 8.6 S: 65.6 ± 10.3	A: 3.4 ± 1.8 w C: 3.8 ± 1.4 w S: 3.8 ± 1.5 w	RCT	0.057	30	M1	FMM
Allman et al. 2016	24	63.5 ± 11.5	54.1 ± 36.3 m	RCT	0.029	9	M1	FMA-UE
Triccas et al. 2015	22	63.4 ± 12.0	19.6 ± 25.7 m	RCT	0.029	18	M1	FMA
Fleming et al. 2017	24	59.8 ± 13.1	19.7 ± 27.4 m	Crossover	0.04	1	M1	JTT
Sattler et al. 2015	20	65.2 ± 11.3	5.5 ± 3.2 d	RCT	0.034	5	M1	JHFT
Saeys et al. 2015	31	63.2 ± 8.7	42.1 ± 18.9 d	Crossover	0.043	16	M1	The Tinetti test
Picelli et al. 2015	20	A: 62.8 ± 11.8 S: 61.0 ± 7.2	A: 51.9 ± 41.1 m S: 54.8 ± 32.9 m	RCT	0.057	10	M1	6MWT
Chang et al. 2015	24	62.8 ± 10.6	16.3 ± 5.6 d	RCT	0.28	10	M1	FMA-LE
Ang et al. 2015	19	54.1 ± 10.6	1037 ± 598 d	RCT	NA	10	M1	FMM
Fusco et al. 2014	16	60.4 ± 14.9	50.9 ± 20.2 d	Crossover	0.043	1	M1	9HPT velocity
Sohn et al. 2013	11	58.45 ± 14.55	63.00 ± 17.27 d	Crossover	0.08	1	M1	Isometric peak torque for knee extensor
Geroin et al. 2011	20	A: 63.7 ± 6.7 S: 63.3 ± 6.4	A: 25.7 ± 6.0 m S: 26.7 ± 5.1 m	RCT	0.043	10	M1	10MWT
Fusco et al. 2014	11	58.36 ± 14.35	19.09 ± 8.04 d	RCT	0.043	10	M1	FMA-UE/6MWT
Klomjai et al. 2018	19	57.2 ± 12.3	3.2 ± 1.7 m	Crossover	0.057	1	M1	FTSTS
Hamoudi et al. 2018	36	A: 61.9 ± 12.7 S: 61.6 ± 12.7	A: 47.9 ± 80.6 m S: 43.7 ± 50.9 m	RCT	0.04	5	M1	JTT

A: anodal tDCS; S: sham tDCS; C: cathodal tDCS; *N*: analyzed sample size; FA: finger acceleration; TUG: time up and go; FMM: upper extremity Fugl-Meyer Motor Score; ARAT: Action Research Arm Test; FMA-UE/LE: Fugl-Meyer Assessment-upper/lower extremity; WMFT-FAS: Wolf Motor Function Test-Functional Ability Scale; JTT: Jebsen Taylor Test; MAS: Motor Assessment Scale; JHFT: the Jebsen Taylor Hand Function Test; 6MWT: 6-minute walk test; 9HPT: the 9-hole peg test; 10MWT: 10-minute walking test; FTSTS: Five-Times-Sit-To-Stand; NA: not mentioned. All data are shown as the mean ± standard deviation or median (range).

**Table 2:** Quality appraisal of the included studies.

Study	1	2	3	4	5	6
Kim et al. 2009	1	1	1	2	0	0
Menezes et al. 2018	1	2	1	2	2	NA
Tahtis et al. 2014	1	2	1	2	0	0
Bolognini et al. 2011	1	2	1	2	0	0
Kwon et al. 2016	1	2	1	2	0	0
Rabadi et al. 2017	1	2	1	2	0	1
Pavlova et al. 2017	1	1	1	2	0	0
Mazzoleni et al. 2017	1	1	1	2	0	0
Figlewski et al. 2017	1	0	1	2	0	4
Takeuchi et al. 2012	1	1	1	2	0	0
Marquez et al. 2017	1	2	1	2	0	0
Straudi et al. 2016	1	2	1	2	0	4
Kim et al. 2010	1	1	1	2	2	2
Goodwill et al. 2016	1	2	1	2	0	0
Hesse et al. 2011	1	2	1	2	1	2
Allman et al. 2016	1	2	1	2	2	NA
Triccas et al. 2015	1	2	1	2	1	7
Fleming et al. 2017	1	1	1	2	1	1
Sattler et al. 2015	1	2	1	2	0	1
Saeys et al. 2015	1	2	1	2	0	0
Picelli et al. 2015	1	2	1	2	0	0
Chang et al. 2015	1	2	1	2	0	NA
Ang et al. 2015	1	0	1	2	0	NA
Fusco et al. 2014	1	2	1	2	2	3
Sohn et al. 2013	1	0	1	2	0	NA
Geroin et al. 2011	1	0	1	2	0	0
Fusco et al. 2014	1	2	1	2	3	1
Klomjai et al. 2018	1	2	1	2	0	3
Hamoudi et al. 2018	1	1	1	2	2	0

1: randomly assigned; 2: blind process; 3: baseline data description; 4: control study; 5: dropouts; 6: side effect; NA: not mentioned.
